# Presence of Antibodies against Genogroup VI Norovirus in Humans

**DOI:** 10.1186/1743-422X-10-176

**Published:** 2013-06-04

**Authors:** João Rodrigo Mesquita, Verónica P Costantini, Jennifer L Cannon, Seh-ching Lin, Maria São José Nascimento, Jan Vinjé

**Affiliations:** 1 Department of Biological Sciences, Faculty of Pharmacy, University of Porto, Porto, Portugal; 2 Agrarian Superior School, Polytechnic Institute of Viseu, Viseu, Portugal; 3 Division of Viral Diseases, National Center for Immunization and Respiratory Diseases, Centers for Disease Control and Prevention, Mail Stop G-04, 1600 Clifton Rd, Atlanta, GA 30333, USA; 4 Center for Food Safety, University of Georgia, Griffin, GA, USA

## Abstract

**Background:**

Noroviruses are important enteric pathogens in humans and animals. Recently, we reported a novel canine norovirus (CaNoV) in dogs with diarrhea belonging to a new genogroup (GVI). No data are available on exposure of humans to this virus.

**Methods:**

Sera from 373 small animal veterinarians and 120 age-matched population controls were tested for IgG antibodies to CaNoV by a recombinant virus like particle based enzyme-linked immunosorbent assay.

**Results:**

Antibodies to CaNoV were found in 22.3% of the veterinarians and 5.8% of the control group (p < 0.001). Mean corrected OD_450_ values for CaNoV antibodies were significantly higher in small animal veterinarians compared to the control group.

**Conclusions:**

These findings suggest that CaNoV may infect humans and small animal veterinarians are at an increased risk for exposure to this virus. Additional studies are needed to assess if this virus is able to cause disease in humans.

## Introduction

Noroviruses (NoVs) are the leading cause of epidemic and sporadic acute gastroenteritis in humans with worldwide an estimated 1 million hospitalizations and up to 200,000 deaths in children < 5 years of age each year [1,2]. Outbreaks occur in various settings including long-term care facilities, hospitals, schools, restaurants and cruise ships. The main modes of transmission of NoV are person-to-person and through the consumption of contaminated food or water. During outbreaks, however, multiple transmission routes may play a role [3]. In recent years NoVs have been detected in a number of mammalian species and several studies have suggested that zoonotic transmission from animal to humans may occur [4-6] and that an animal reservoir might be the source of the introduction of new strains in the human population. Although no zoonotic events have been reported, there are several indications that NoVs may be able to cross the species barrier. Gnotobiotic pigs have been experimentally infected with a human NoV strain [4], and viruses closely related to human NoVs have been detected in swine [7]. Moreover, NoV sequences have been detected in livestock and in retail meat samples highlighting a possible route for indirect zoonotic transmission of NoVs through the food chain and the risk for emergence of animal/human recombinants [8].

Noroviruses are a group of non-enveloped, single-stranded, RNA viruses with an icosahedral capsid symmetry classified into the genus *Norovirus* of the family *Caliciviridae*. They can be grouped in at least 5 different genogroups (designated GI-GV) [1,9]. Strains infecting humans are found in GI, GII and GIV. Porcine NoVs are classified in distinct genotypes within GII, bovine and ovine viruses belong uniquely to GIII, and murine NoVs are grouped in GV. Recently, several research groups have reported NoVs in domestic carnivores with diarrhea [10,11]. Canine NoVs (CaNoVs) genetically related to GIV have been reported in Italy, Greece and Japan [10-13], whereas viruses belonging to a proposed new genogroup (GVI) were found in fecal samples from dogs with diarrhea in Portugal and Italy [9,14-16].

The zoonotic potential of an infectious disease agent has been inferred by comparing pathogen-specific antibody levels between individuals that are in close contact with a particular animal and a matched control population with no professional exposure to animals [17,18]. For example, a higher serum antibody level against bovine NoV was detected in large animal veterinarians compared to the general population, indicating that bovine NoV strains could infect the human population [18]. Additionally, antibodies to human NoVs have been detected in pigs highlighting the possibility of human-to-animal transmission of NoV [5].

In most industrialized countries, pets are an integral part of the household leading to well-documented health risks associated with owning a pet. Bites, scratches and allergies are more frequent; however, infections including parasitic, bacterial, fungal and viral diseases can be transmitted to humans [19]. In a recent report, human NoV sequences were detected in fecal samples from pet dogs which had been in direct contact with humans with NoV gastroenteritis, suggesting that human NoVs can at least survive in the gastrointestinal tract of dogs [20].

To investigate if CaNoV may infect humans, sera from pet veterinarians and age-matched population controls were tested for IgG antibodies to recombinant virus-like particles of CaNoV. This Study Protocol was published elsewhere [21].

## Results

**Figure 1:**
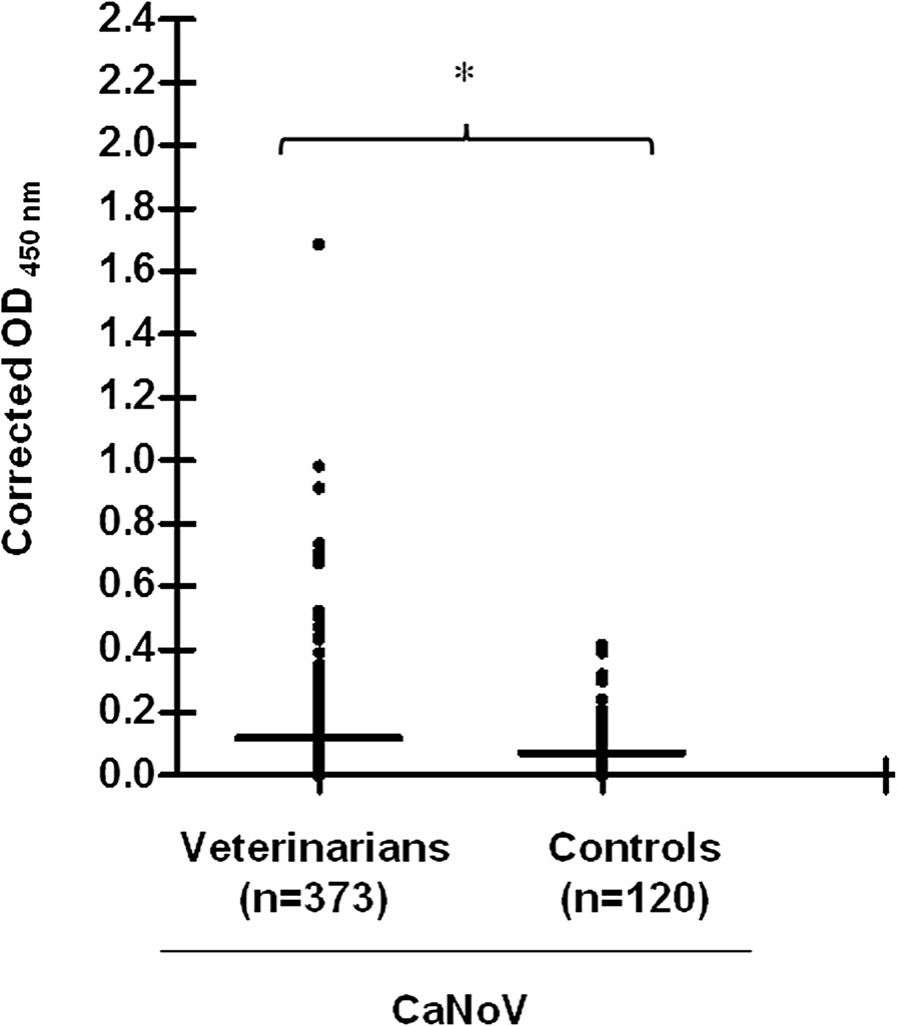
**Corrected optical densities [OD] at 450 nm for canine norovirus antibodies in sera from veterinarians and controls.** Sera were tested for the presence of CaNoV antibodies in a VLP-based ELISA at 1:1,500 dilution. Values are the corrected optical densities (OD) at 450 nm [OD_450_ (VLP coated) - OD_450_ (non-coated)] for each serum sample and the mean corrected OD_450_ value of each group (horizontal bars). Groups were compared and analyzed by Mann–Whitney U-test. Differences were considered significant (*) at p < 0.05.

Of the 373 veterinarians, 83 (22.3%) had IgG antibodies against CaNoV compared to 7 (5.8%) of the 120 matched population controls (p < 0.001). Moreover, the mean corrected OD_450_ values for CaNoV antibodies was significantly higher in veterinarians than in controls (p < 0.001) (Figure 1). CaNoV antibodies were detected in veterinarians from all four countries.

**Figure 2:**
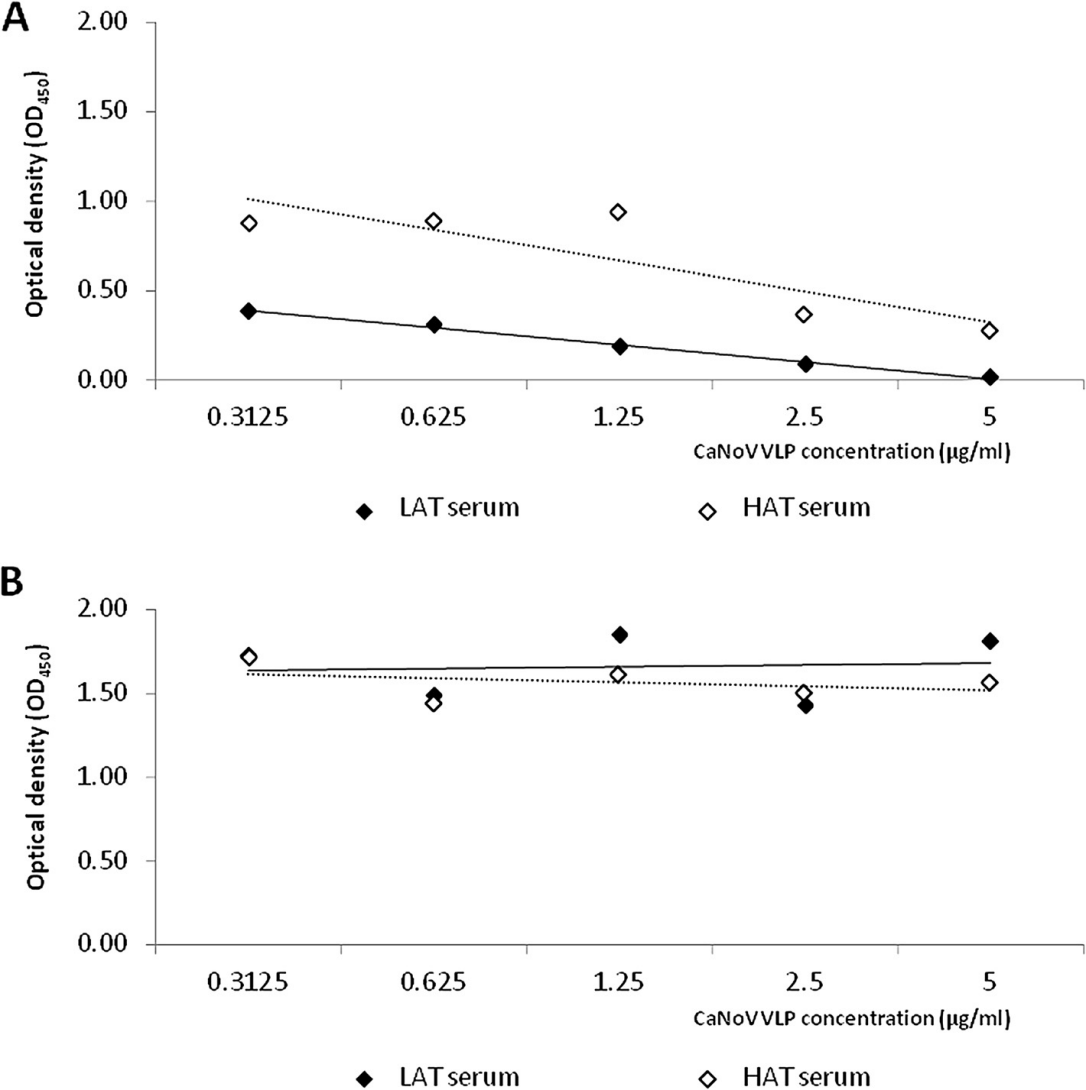
**Evaluation of potential cross-reactivity between human GII.4 NoV IgG antibodies and canine NoV VLPs.** Two sera with low (LAT) and high (HAT) CaNoV antibody titer were pre-incubated with a 2-fold serial dilution of CaNoV VLPs (5, 2.5, 1.25, 0.625, 0.3125 μg/ml) and tested for antibodies against CaNoV VLPs (**A**) and human GII.4 NoV VLPs (**B**). Values are the corrected optical densities (OD) at 450 nm [OD_450_ (VLP coated) - OD_450_ (non-coated)] for each serum sample. Dotted lines represent the logistic regression of HAT values, solid lines represent the logistic regression of LAT values.

To evaluate possible cross-reactivity between these antibodies and human NoV, two serum samples from veterinarians with high (HAT) and low antibody titers (LAT) to CaNoV were pre-incubated with 2-fold serial dilutions of CaNoV VLPs. The corrected OD_450_ values for CaNoV antibodies in both serum samples decreased significantly with increasing concentration of CaNoV VLPs (β = −0.150 ± 0.043, 95%CI −0.287 to −0.013, p < 0.05 and β = −0.073 ± 0.018, 95%CI −0.132 to −0.014, p <0.05 for serum HAT and LAT, respectively) (Figure 2A). By contrast, no significant change in the corrected OD_450_ values was observed when the pre-incubated sera were tested for the presence of GII.4 New Orleans antibodies (β = −0.011 ± 0.031; 95%CI −0.11 to 0.089 and β = 0.0219 ±0.056; 95%CI 0.158 to 0.202 for samples 68 and 25, respectively) (Figure 2B).

## Discussion

We detected IgG antibodies against CaNoV in 22.3% of the small animal veterinarians and in 5.8% of the age-matched population controls. These findings suggest that CaNoV may infect humans and that small animal veterinarians are at an increased risk for exposure and possibly infection with this virus. The presence of antibodies in the population control samples may be explained by household contact as dogs are popular pet animals.

An increased exposure risk to bovine NoV has been reported for large animal veterinarians in the Netherlands who had a higher seroprevalence of bovine NoV antibodies than the general population [18]. Conversely, a high prevalence of antibodies against human NoV (Norwalk strain) was detected in pigs and captive juvenile macaques [5,22]. These data suggest that NoV may be able to cross the species barrier. Exposure to zoonotic agents is a widely recognized risk in veterinary medicine. In an Australian survey, 4% of veterinarians reported having acquired at least one zoonotic disease from animal-related exposure [23]. In another study, IgG antibodies against *Brucella* spp and *Coxiella burnetii* were higher among veterinarians working in an endemic region [24]. The zoonotic risk of veterinarians and pig handlers for hepatitis E virus (HEV) infections has been demonstrated in studies in Taiwan and the US [25,26]. In the Taiwan study, 27% of pig handlers tested positive for anti-HEV antibodies compared to 8% of the control subjects [25], while in the US study 26% of veterinarians working with swine and 17% of blood donors were seropositive for HEV, suggesting that veterinarians may be at a higher risk of HEV infection through animal contact, compared to normal blood donors [26].

A limitation of our study was that the antibodies detected by the CaNoV VLPs may be cross-reactive against human NoVs. However, the blocking assay data showed that binding of CaNoV antibodies but not human NoV antibodies, could be blocked by CaNoV VLPs, demonstrating the VLP-based ELISA used in this study measured CaNoV-specific antibodies.

In conclusion, our data suggest that CaNoV may infect humans and that small animal veterinarians are at an increased risk. Studies that test human stool samples in households with dogs with CaNoV diarrhea are needed to confirm that this virus is able to cause diarrheal disease in humans.

## Materials and methods

### Serum samples

A total of 373 pet veterinarians from four different countries (Portugal, Spain, Brazil, and United Kingdom) who attended the Annual Veterinary Meeting in January 2012 in Santa Maria da Feira, Portugal, were enrolled in the study after giving informed consent (Table 1). Blood was obtained by venipuncture from all enrolled veterinarians. In addition, 120 sera matched by age (in 5-year age groups) and sex were collected from anonymous volunteers from the University of Porto. This study was approved by the institutional review board at the University of Porto (Parecer n°18/CEUP/2011).

**Table 1:** D**escriptive epidemiology of 373 veterinarians participating in the study**

	**% (N**^**1**^**)**	**95%CI**^**2**^
Age		
19-29	51.7 (193)	46.7-56.8
30-39	38.8 (145)	33.9-43.8
40-49	7.8 (29)	5.1-10.5
≥50	1.7 (6)	0.3-2.9
Sex		
Female	70.5 (263)	65.9-75.1
Male	29.5 (110)	24.9-34.1
Residence		
Foreign^3^	6.4 (24)	3.9-8.9
Portugal	93.6 (349)	91.1-96.1
Years in practice		
1-10	80.2 (299)	76.1-84.2
11-20	16.1 (60)	12.4-19.8
>20	3.7 (14)	1.8-5.7

^1^Number of individuals in each category; ^2^Confidence Interval; ^3^Foreign: residents of Brazil, Spain or United Kingdom.

### Canine norovirus VLP-based antibody ELISA

**Figure 3:**
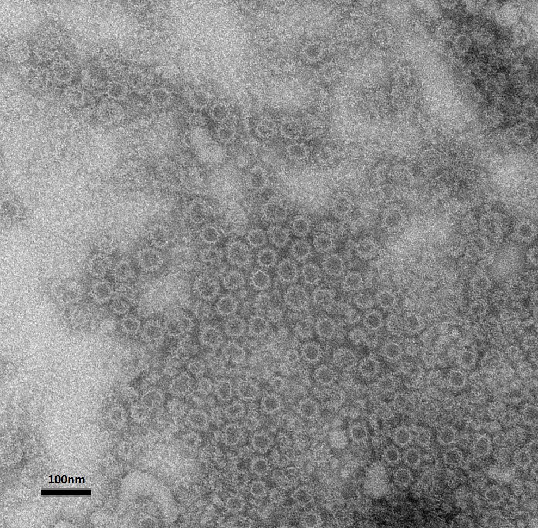
**Electron microscopy of CaNoV VLPs confirming a norovirus-like morphology and size.** Grids were stained with 2% potassium phosphotungstate.

Recombinant virus-like particles (VLPs) of CaNoV [dog/C33/Viseu/2007/PRT, GenBank accession number: GQ443611.1] were produced by cloning full-length VP1/VP2 (ORF2 and ORF3 of the genome) in a baculovirus-insect cell expression system. Recombinant VLPs were recovered from the culture media and purified through sucrose and CsCl gradients [27]. Norovirus morphology and size of the purified VLPs was confirmed by electron microscopy (Figure 3).

Canine NoV VLPs (0.25 μg per well) were coated into 96-well microtiter plates (NUNC, Milford, USA) in carbonate–bicarbonate buffer (0.01 M, pH 9.6), and incubated overnight at 4°C. Coated plates were washed with PBS/0.5% Tween-20 and blocked with PBS/0.5% Tween 20/ 5% non-fat dry milk (blocking buffer) for 2 h at 37°C. Serum samples were diluted 1:1,500 in blocking buffer and tested in duplicate in VLP-coated and non-coated wells, to correct for sample background. After 1 hour incubation at 37°C, bound IgG was detected by peroxidase-labeled goat anti-dog IgG (H+L) (1:12,800) and TMB substrate (Kirkegaard & Perry Laboratories, Gaithersburg, USA). Background signal [OD_450 (non-coated wells)_] was subtracted from each sample to obtain a corrected OD_450_ [OD_450 (VLP coated)_ - OD_450 (non-coated)_]. Cut off value of the test was established as the mean of the OD_450 (non-coated wells)_ plus 3 standard deviations (3SD). A serum sample was considered positive when the corrected OD_450_ was higher than the cut off.

### Blocking assay

Two serum samples with high (HAT) and low antibodies levels (LAT) against CaNoV and high levels of anti GII.4 New Orleans antibodies were pre-incubated with 2-fold serial dilutions of CaNoV VLPs (5, 2.5, 1.25, 0.625, 0.3125 μg/ml) for 1 h at 37°C. After incubation, 50 μL of pre-incubated sera were tested in duplicate for the presence of CaNoV antibodies as described above, and for the presence of human NoV antibodies using GII.4 New Orleans VLPs [Hu/GII.4/New Orleans1805/2009/USA, GenBank accession number: GU445325.2] with slight modifications (coating with 0.0625 μg per well and detection of bound IgG by peroxidase-labeled goat anti-human IgG (H+L) (1:12,800)).

### Statistical analysis

A χ2 test for unequal odds with Yates’ continuity correction was used to determine significant differences in CaNoV prevalence between study groups. Mann–Whitney U-test was used to assess differences in CaNoV antibody magnitude between study groups. P values less than 0.05 were considered statistically significant. Statistical analyses were performed with R software [28].

## Competing interests

The authors declare that they have no competing interests.

## Authors’ contributions

JM conceived the study, collected the sera, carried out the immunoassays and drafted the manuscript. VC helped design the immunoassays, and helped draft the manuscript. JC cloned and sequenced the canine norovirus strain. SL produced the virus-like particles. MSJN conceived the study and participated in the design of the study. JV participated in the design and coordination of the study and drafted the manuscript. All authors read and approved the final manuscript.

## References

[B1] Glass RI Parashar UD Estes MK Norovirus gastroenteritis N Engl J Med 2009 361 1176 1185 10.1056/NEJMra0804575 PMC3880795 19864676

[B2] Lopman B Gastanaduy P Park GW Hall AJ Parashar UD Vinje J Environmental transmission of norovirus gastroenteritis Curr Opin Virol 2012 2 96 102 10.1016/j.coviro.2011.11.005 22440972

[B3] Becker KM Moe CL Southwick KL MacCormack JN Transmission of Norwalk virus during football game N Engl J Med 2001 26 1223 1227 10.1056/NEJM200010263431704 11071673

[B4] Cheetham S Souza M Meulia T Grimes S Han MG Saif LJ Pathogenesis of a genogroup II human norovirus in gnotobiotic pigs J Virol 2006 80 10372 10381 10.1128/JVI.00809-06 17041218 PMC1641747

[B5] Farkas T Nakajima S Sugieda M Deng X Zhong W Jiang X Seroprevalence of noroviruses in swine J Clin Microbiol 2005 43 657 661 10.1128/JCM.43.2.657-661.2005 15695660 PMC548037

[B6] Bank-Wolf BR König M Thiel HJ Zoonotic aspects of infections with noroviruses and sapoviruses Vet Microbiol 2010 140 204 212 10.1016/j.vetmic.2009.08.021 19773133

[B7] Wang QH Han MG Cheetham S Souza M Funk JA Saif LJ Porcine noroviruses related to human noroviruses Emerg Infect Dis 2005 11 1874 1881 10.3201/eid1112.050485 16485473 PMC3367634

[B8] Mattison K Shukla A Cook A Pollari F Friendship R Kelton D Bidawid S Farber JM Human noroviruses in swine and cattle Emerg Infect Dis 2007 13 1184 1188 10.3201/eid1308.070005 17953089 PMC2828081

[B9] Mesquita JR Barclay L Nascimento MS Vinje J Novel norovirus in dogs with diarrhea Emerg Infect Dis 2010 16 980 982 10.3201/eid1606.091861 20507751 PMC3086253

[B10] Martella V Lorusso E Decaro N Elia G Radogna A D’Abramo M Desario C Cavalli A Corrente M Camero M Germinario CA Banyai K Di Martino B Marsilio F Carmichael LE Buonavoglia C Detection and molecular characterization of a canine norovirus Emerg Infect Dis 2008 14 1306 1308 10.3201/eid1408.080062 18680664 PMC2600395

[B11] Martella V Pinto P Buonavoglia C Canine noroviruses Vet Clin N Am-Small 2011 41 1171 1181 10.1016/j.cvsm.2011.08.002 PMC7114684 22041209

[B12] Ntafis V Xylouri E Radogna A Buonavoglia C Martella V Outbreak of canine norovirus infection in young dogs J Clin Microbiol 2010 48 2605 2608 10.1128/JCM.02528-09 20484606 PMC2897520

[B13] Tse H Lau SK Chan WM Choi GK Woo PC Yuen KY Complete genome sequences of novel canine noroviruses in Hong Kong J Virol 2012 86 9531 9532 10.1128/JVI.01312-12 22879606 PMC3416109

[B14] Martella V Decaro N Lorusso E Radogna A Moschidou P Amorisco F Lucente MS Desario C Mari V Elia G Banyai K Carmichael LE Buonavoglia C Genetic heterogeneity and recombination in canine noroviruses J Virol 2009 83 1391 1396 10.1128/JVI.01385-09 PMC2772758 19710153

[B15] Mesquita JR Nascimento MS Molecular epidemiology of canine norovirus in dogs from Portugal, 2007–2011 BMC Vet Res 2012 8 107 10.1186/1746-6148-8-107 22776749 PMC3410785

[B16] Mesquita JR Nascimento MS Gastroenteritis outbreak associated with faecal shedding of canine norovirus in a Portuguese kennel following introduction of imported dogs from Russia Transbound Emerg Dis 2012 59 456 459 10.1111/j.1865-1682.2011.01284.x 22151979

[B17] Pedersden KA Sadasiv EC Chang PW Yates VJ Detection of antibody to avian viruses in human populations Epidemiol Infect 1990 104 519 525 10.1017/S095026880004752X 2161349 PMC2271774

[B18] Widdowson MA Rockx B Schepp R van der Poel WH Vinje J van Duynhoven YT Koopmans MP Detection of serum antibodies to bovine norovirus in veterinarians and the general population in the Netherlands J Med Virol 2005 76 119 128 10.1002/jmv.20333 15779045

[B19] Chomel BB Sun B Zoonoses in the bedroom Emerg Infect Dis 2011 17 167 172 10.3201/eid1702.101070 21291584 PMC3298380

[B20] Summa M von Bonsdorff CH Maunula L Pet dogs–a transmission route for human noroviruses? J Clin Virol 2012 53 244 247 10.1016/j.jcv.2011.12.014 22244255

[B21] Mesquita JR Nascimento MSJ Serosurvey of veterinary conference participants for evidence of zoonotic exposure to canine norovirus – study protocol Virol J 2012 9 250 10.1186/1743-422X-9-250 23110789 PMC3495783

[B22] Farkas T Dufour J Jiang X Sestak K Detection of norovirus-, sapovirus- and rhesus enteric calicivirus-specific antibodies in captive juvenile macaques J Gen Virol 2010 91 734 738 10.1099/vir.0.015263-0 19889933 PMC2888095

[B23] Jeyaretnam J Jones H Phillips M Disease and injury among veterinarians Aust Vet J 2000 78 625 629 10.1111/j.1751-0813.2000.tb11939.x 11022291

[B24] Ergonul O Zeller H Kilic S Kutlu S Kutlu M Cavusoglu S Esen B Dokuzoguz B Zoonotic infections among veterinarians in Turkey: Crimean-Congo hemorrhagic fever and beyond Int J Infect Dis 2006 10 465 469 10.1016/j.ijid.2006.06.005 16978897

[B25] Hsieh SY Meng XJ Wu YH Liu ST Tam AW Lin DY Liaw YF Identity of a novel swine hepatitis E virus in Taiwan forming a monophyletic group with Taiwan isolates of human hepatitis E virus J Clin Microbiol 1999 37 3828 3834 10565892 10.1128/jcm.37.12.3828-3834.1999 PMC85823

[B26] Meng XJ Wiseman B Elvinger F Guenette DK Toth TE Engle RE Emerson SU Purcell RH Prevalence of antibodies to hepatitis E virus in veterinarians working with swine and in normal blood donors in the United States and other countries J Clin Microbiol 2002 40 117 122 10.1128/JCM.40.1.117-122.2002 11773103 PMC120098

[B27] Jiang X Wang M Graham DY Estes MK Expression, self-assembly, and antigenicity of the Norwalk virus capsid protein J Virol 1992 66 6527 6532 1328679 10.1128/jvi.66.11.6527-6532.1992 PMC240146

[B28] R Development Core Team R: A language and environment for statistical computing 2010 Vienna, Austria: R Foundation for Statistical Computing ISBN 3-900051-07-0, URL http://www.R-project.org/

